# Cross-cultural adaption, translation and validation of the Toronto extremity salvage score (TESS) for patients in German-speaking countries

**DOI:** 10.1007/s00508-021-01865-4

**Published:** 2021-04-27

**Authors:** Carmen Trost, Christoph Hofer, Tanja Stamm, Reinhard Windhager, Gerhard M. Hobusch

**Affiliations:** 1 Department of Orthopedics and Trauma Surgery, Division of Orthopedics, Medical University of Vienna, Vienna, Austria; 2 Institute for Outcomes Research, Medical University of Vienna, Vienna, Austria

**Keywords:** German, TESS, Limb reconstruction, Bone sarcoma, Soft tissue sarcoma, Quality of life, Questionnaire, Sarcoma

## Abstract

**Objective:**

The preferred treatment for malignant bone and soft tissue tumors is limb salvage surgery; the Toronto extremity salvage score (TESS) is commonly used to measure physical functioning of the affected extremity. The aims of this study were to translate and culturally adapt the German version of the TESS, as well as to explore its convergent reliability, validity and re-test reliability.

**Study design:**

Patients (*n* = 50) 32 lower extremity (LE) and 18 upper extremity (UE) were asked to fill out the German TESS two times (t1: clinical visit, t2: regular email) and the SF-36 once.

**Methods:**

The TESS questionnaires were translated from English into German, back translated into English, and culturally adapted. The reliability was assessed with Cronbach’s alpha (α). The validity was measured with the SF-36 physical component score and TESS using the Spearman rank correlation coefficient (r). Furthermore, the test-retest reliability was calculated with the intraclass correlation coefficient (ICC).

**Results:**

Internal consistency for both questionnaires was excellent (LE t1: α = 0.924, t2: α = 0.952; UE t1: α = 0.957, t2: α = 0.898). A statistically significant correlation was found between the SF-36 physical component scale and the German TESS (LE r = 0.741, UE r = 0.713). The ICC between baseline (t1) and re-test (t2) was 0.952 and 0.871 for the lower and upper extremities, respectively.

**Conclusion:**

Initial evidence demonstrated that the German TESS is a valid and reliable instrument for use with patients after surgical treatment of malignant bone or soft tissue sarcoma.

**Supplementary Information:**

The online version of this article (10.1007/s00508-021-01865-4) contains supplementary material, which is available to authorized users.

## Introduction

The treatment of choice for malignant bone and soft tissue tumors in the extremities is limb salvage surgery [1]. Nearly two decades ago, the Toronto extremity salvage score (TESS), a patient-reported outcome score, was added to the previously developed clinician-reported Musculoskeletal Tumor Society Score (MSTS) for use in musculoskeletal research and in the routine care of oncology patients to assess outcomes [1–3]. This valid and reliable instrument was developed to measure physical functioning in daily life by assessing disability at multiple time points and possible changes in individuals’ movement [4]. Furthermore, it is intended and suitable for evaluating treatment success [1, 4].

The TESS is commonly used in several countries. The original instrument was developed in English and has been translated into the following languages: Dutch [5], Japanese [6], Korean [7], Danish [8], Portuguese [9], Chinese [10], Finnish [11] and Italian [12]. To date the TESS has not been translated into German for use in German-speaking countries, which represent a large portion of European patients. A validated, accurate and precise instrument is urgently needed for benchmarking, value-based health care, international research collaborations and cross-border care. Therefore, this manuscript aims to establish and validate a culturally sensitive German version of the TESS.

## Methods

We conducted a psychometric study consisting of the translation and cultural adaptation of the Toronto extremity salvage score (TESS) into German, and analysis of the validity, reliability and internal consistency of the underlying instrument [13, 14]. The ethics committee of the local university approved and reviewed this study. Every participant signed an informed consent form.

### Sample

Patients from the Orthopedic Sarcoma Outpatient Clinic of the Department of Orthopedics and Trauma Surgery Vienna were asked to participate in this study from October 2018 to April 2019. Patients were asked to participate if they met the following inclusion criteria: i) 18 years or older; ii) diagnosed with sarcoma of the upper or lower extremities; iii) at minimum 3 months post-limb salvage surgery; and iv) no disease recurrence or other serious disease in the past. Patients unable to fill out the questionnaire on their own, e.g. did not speak German, were not asked to complete the questionnaire. Both the TESS and the SF-36 were administered during the waiting time in a separate, quiet room to preserve privacy and create a calm atmosphere. After finishing, patients were asked to fill out the TESS test again alone at home and send it back to the clinic. In summary, 50 patients completed the questionnaires, 18 for the upper extremity and 32 for the lower extremity. Response rates for the at-home evaluation were 77.8% (*n* = 14) and 62.5% (*n* = 20) for the upper and lower extremity groups, respectively. If a questionnaire was not returned on time, the authors called the patient and sent the questionnaire again; after the second reminder and no response or a negative response, it was noted in the source data that the patient did not or would not send back the questionnaire. Primarily the validation and reliability of the test was examined, so the authors decided not to differ between the evaluation time points asked on the title page of the TESS.

### Translation and cross-cultural adaption

**Fig. 1:**
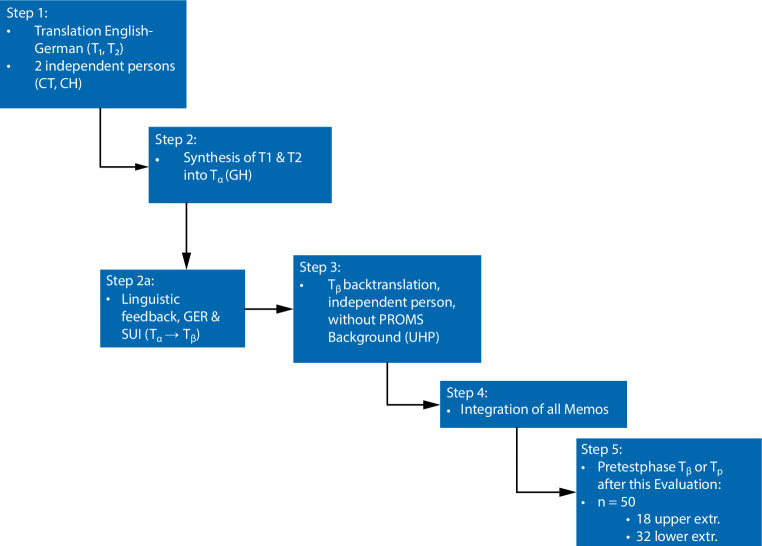
Schematic diagram of the interactive translation and cross-cultural adaption process (the translation and cross-cultural adaption process were modified from Beaton et al. and Wild et al. [3, 4, 11])

Five fundamental steps of translation and adaption were carried out for the intercultural adjustment process (Fig. 1; [3, 4]): (1) translation of the original version into the target language; (2) synthesis of both translations; (2a) linguistic feedback; (3) back translation by an independent translator; (4) discussion and approval by the authors and (5) pretesting and evaluation. In the first step (1), two independent persons translated the original English language TESS, resulting in two separate transcripts with a third for comparison. The translators were German native speakers fluent in English with university level experience in the language: a sociologist (CT), a medical student (CH), and a professor of statistics and outcome measures (TS, the comparison version) [9]. Second (2), the orthopedic surgeon (GH), who was not included in the initial process, merged the two translations. Between steps two and three (2a), two German-speaking individuals were interviewed for linguistic feedback. Third (3), the back translation was performed by a blinded independent translator without a medical background and with no further interaction in this process. Fourth (4), the committee, composed of the authors—surgeon (GH), patient reported outcome measurement scores (PROMS) scientist (TS), sociologist (CT), and medical student (CH)—and a translation expert, discussed all transcripts, translations and comments, and decided on the final wording of the items. These were then used for the pretest phase (5). Six patients with sarcoma in the upper or lower extremities were randomly selected then interviewed by two authors; one conducted the interview and the other observed and took notes. The direct translations for two questions were too long and confusing for the pretest patients to answer without support from the interviewer [15–17]. Therefore, these questions (UE 25, 26 and LE 26, 27) were changed so the TESS can be completed by patients themselves without any assistance from or explanation of questions by healthcare providers (12–14). Furthermore, because distinct transcripts from two authors (CH and CT) were merged into one final version during the translation process, minor changes were made to most questions. See Table 1 for further details: “wording shortened”—the length of the question was reduced; “word changed”—the wording was simplified; “sentence structure”—the sentence structure was changed; and “equal”—the translations for upper and lower extremities were equivalent and the question was the same in the upper and lower TESS German versions. Lastly, Swiss-German and German-German native speakers were involved to minimize language barriers and adjust for different dialects. This process resulted in the final version of the German TESS. The SF-36 was used to anchor the validity assessment.

**Table 1:** Translation and cultural adaption results table

	UE			LE		
QU	Changes	Swiss	German	Changes	Swiss	German
1	Wording shortened	–	–	Equal	–	–
2	Word changed	–	–	Equal	–	–
3	Sentence structure		–	Word changed	Comment; not necessary (socks/knee socks)	–
4	T1 + T2	–	–	Equal	–	–
5	T2	–	–	Equal	–	–
6	Word changed	–	–	Equal	–	–
7	Word changed/sentence structure	–	–	Equal	Comment; not necessary (meals/eat)	–
8	Word changed/sentence structure	–	–	T1 + T2	–	–
9	T1 + T2	–	–	T2	–	–
10	T1 + T2	–	–	Word changed/sentence structure	–	–
11	T2	–	–	Word changed/sentence structure	–	–
12	Wording shortened	–	–	T2	Word changed: chair	–
13	Wording shortened	–	–	Wording shortened	–	–
14	T2	–	–	Word changed	–	–
15	T1 + T2	–	–	Wording shortened	–	Word changed: stairs
16	T2	–	–	Wording shortened	–	Word changed: stairs
17	T1 + T2	–	–	T1 + T2	–	–
18	Word changed/sentence structure	–	–	Wording shortened	–	–
19	T2	–	–	Wording shortened	–	–
20	Word changed/sentence structure	–	–	T1 + T2	–	–
21	T1	–	–	Wording shortened	–	–
22	T2	Word changed: open	–	T1 + T2	–	–
23	T1 + T2	–	–	T2	–	–
24	Word changed	–	–	Word changed/sentence structure	–	–
25	Word changed/sentence structure	Comment; not necessary (job/work)	–	Sentence structure	–	–
26	Word changed/sentence structure	–	–	Equal	–	–
27	Word changed/sentence structure	–	–	Equal	–	–
28	T1 + T2	–	–	Equal	–	–
29	Word changed/sentence structure	Sports activities; Swiss: hobbies	–	Equal	–	–
30	–	–	–	Word changed/sentence structure	–	–
1	Word changed/sentence structure	–	–	Equal	–	–
2	Word changed	–	–	Equal	–	–

*T1* author CH, *T2* author CT, synthesis authors GH and TS

At the baseline assessment, patients were asked to fill out the SF-36 first, followed by the lower or upper TESS questionnaire. For the second assessment, patients were instructed to fill out the lower or upper TESS alone at home 1 week after baseline and to send it back to the clinic. A gap of 1 week was planned so patients would not have forgotten the entire procedure but would not remember each of their answers. It was also needed for administrative tasks and planning the individual steps for adherence [7].

## Statistics

### Validity

Spearman rank correlation coefficients were calculated between the SF-36 dimension summary scale scores and the TESS scores. The SF-36 is a commonly used instrument for evaluating aspects of health-related quality of life and functioning [13, 18, 19].

### Reliability

Cronbach’s alpha was used to determine internal consistency [14]. For the test-retest analysis, the intraclass correlation coefficient (ICC) was calculated between baseline and repetition for responses to each question and for the total TESS score [20]. The sample size calculation was based on the reliability analysis: for an expected ICC of 0.89, a confidence interval of 0.23, and with 2 measurements per individual, a sample size of 14 individuals was required [5–11]. Bland-Altman plots were used to visually assess the fluctuation range of the deviations [21].

The statistical analyses were conducted using the Statistical Package for the Social Sciences, Version 25.0. (IBM SPSS, Chicago, IL, USA). For the correlation coefficients and the ICC we defined ≥ 0.70 as strong, ≥ 0.50–< 0.70 as moderate, and ≤ 0.50 as weak [5, 7, 18, 19]. A *p*-value of 0.05 was considered statistically significant.

## Results

### Translation and cross-cultural adaption

Two questions (LE 26, 27; UE 25, 26) were shortened and simplified. The linguistic feedback showed that Swiss, German and Austrian persons have different understanding of the words “chair” (Sessel, Stuhl) and “to open a door” (aufziehen/aufmachen), whereas there are no such differences in English. Therefore, words that have the same meaning for Swiss, German and Austrian persons have been used in the German Toronto extremity salvage score (TESS) version.

### Statistics

#### Validity

Correlations between participants and SF-36 physical and mental component scores (PCS/MCS) are listed in Table 2; as expected, the MSC correlation was low. Mean scores for the 8 SF-36 dimensions of the patients are shown in Table 3.

**Table 2:** Construct validity for the German Toronto extremity salvage score (TESS) version, calculated with the Spearman rank correlation

Validity		
Spearman rank correlations of the TESS (upper and lower extremities) with the SF-36 component scores		
Spearman	Lower extremity	Upper extremity
Physical component score	0.741	0.713
Mental component score	0.570	0.277

**Table 3:** Mean and median scores of the Toronto extremity salvage score (TESS) and SF-36 for the lower and upper extremities

	Lower extremity		Upper extremity	
	Mean (SD)	Median (range)	Mean (SD)	Median (range)
TESS	77.6 (19.5)	81.8 (19.4–100.0)	80.8 (14.3)	84.5 (50.9–80.8)
*SF-36*				
Physical functioning	69.7 (22.2)	70 (0.0–100.0)	67.1 (24.0)	70.0 (15.0–100.0)
Role limitations: physical	56.8 (41.1)	75.0 (0.0–100.0)	75.0 (37.3)	100.0 (0.0–100.0)
Social functioning	75.4 (25.9)	87.5 (25.0–100.0)	84.9 (24.5)	87.5 (12.5–100.0)
Role limitations: emotional	65.7 (44.5)	100.0 (0.0–100.0)	80.7 (35.7)	100.0 (0.0–100.0)
Mental health	71.6 (18.0)	80.0 (20.0–100.0)	77.3 (16.3)	80.0 (40.0–100.0)
Vitality	60.5 (16.6)	60.0 (30.0–90.0)	60.3 (13.8)	60.0 (45.0–90.0)
Bodily pain	66.7 (25.4)	62.0 (12.0–100.0)	70.3 (20.8)	72.0 (31.0–100.0)
General health perceptions	64.5 (20.9)	62.0 (20.0–100.0)	62.7 (21.1)	67.0 (0.0–95.0)
Physical component score	43.6 (9.1)	44.6 (31.1–53.4)	46.1 (7.2)	44.0 (18.2–58.1)
Mental component score	46.8 (12.9)	48.5 (36.2–67.0)	53.4 (9.3)	52.4 (11.5–63.5)

### Reliability

**Fig. 2:**
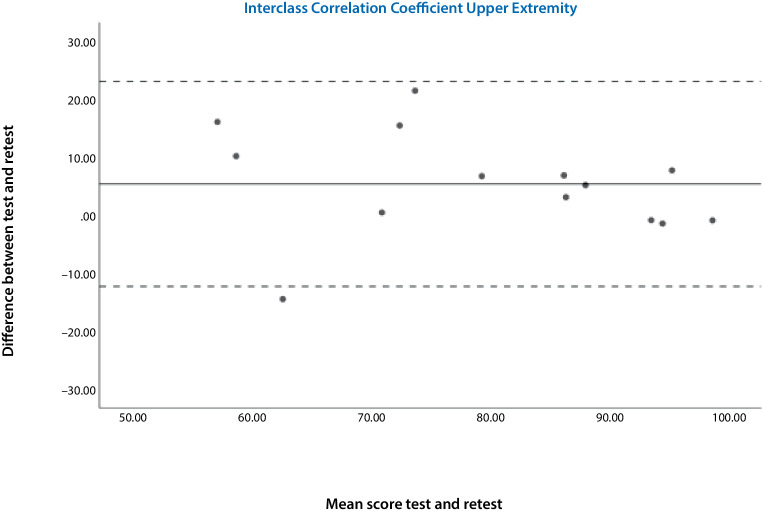
Bland-Altman plots show the results of the test-retest reliability of the upper German Toronto extremity salvage score (TESS) version. The *bold line* shows the mean difference between the two tests (baseline and repetition) and the *dashed lines* represent the 95% confidence interval. The *middle line* shows the fluctuation margin of the deviations. There is only one outlier. This figure shows that there are no signs of systematic bias

**Fig. 3:**
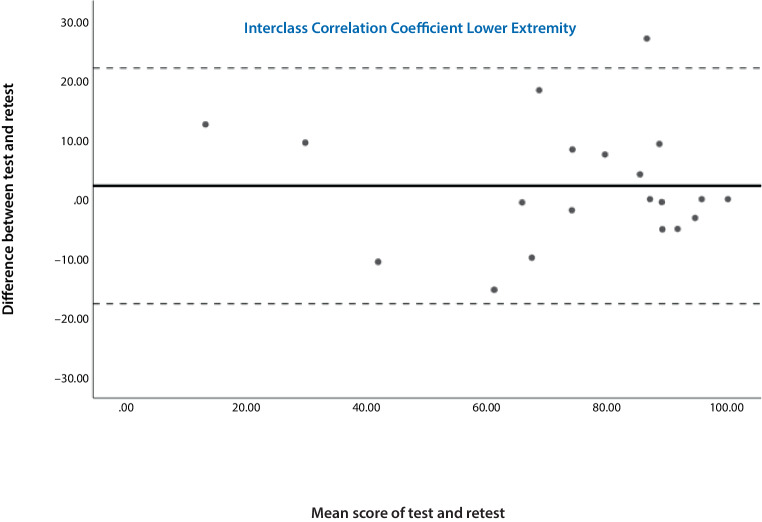
Bland-Altman plots show the results of the test-retest reliability of the lower German Toronto extremity salvage score (TESS) version. The *bold line* shows the mean difference between the two tests (baseline and repetition) and the *dashed lines* represent the 95% confidence interval. The middle line shows the fluctuation margin of the deviations. There is only one outlier. This figure shows that there is no sign of systematic bias

Internal consistency was excellent for both questionnaires (LE t1: α = 0.924, t2: α = 0.952; UE t1: α = 0.948, t2: α = 0.898). This confirmed the homogeneity of all parts of the instrument. The ICC was 0.952 for the lower extremity version and 0.871 for the upper extremity version. The Bland-Altman plots for both questionnaires (Figs. 2 and 3) showed that there were no systematic biases; the points were equally spread around the middle line.

## Discussion

The original English and various language versions of the Toronto extremity salvage score (TESS) questionnaires for both lower and upper extremities are commonly used self-reported outcome measurements for functioning after limb salvage surgery for bone or soft tissue tumors [4–11]; however, to date there is no validated German version. In this study, this instrument was translated and culturally adapted into German versions for three German-speaking countries: Austria, Germany and Switzerland. The cross-cultural adaption and validation in this study was conducted according to internationally accepted guidelines [15–17] and based on the previously translated, adapted and validated TESS versions in other languages [4–11]. During the linguistic feedback process a few discrepancies in wording were identified and some phrases modified accordingly.

The German version demonstrates comparable validity, reliability, internal consistency and test-retest reliability to validated versions of the TESS in other languages [4–11]. The small sample size for the upper extremities is a limitation in this study; however, we calculated the sample size based on the reliability analysis, also considering the rare disease and a steady recruitment process and based on results from the other validated TESS versions [4–11]. A sample size of 14 individuals was required to achieve an expected ICC of 0.89 and a desired confidence interval of 0.23, with 2 measurements per individual. It was not necessary to prolong the study for further recruitment.

As in the validation of the Dutch version, the SF-36 was used [5] rather than the MSTS to test validity [6, 7, 10]. The Musculo Skeletal Tumor Society Score (MSTS) is a clinician-reported outcome measure and is not available in a validated German version, so the patient-reported and well-established SF-36 was used instead. As expected, the mental component score (MCS) showed low (LE 0.570) and no (UE 0.277) significance. Because the TESS is specific to functioning, the physical component score (PCS) was important to explore the validity.

The aim of this study was to translate the TESS questionnaire into German and to adapt it to regional differences between Germany, Austria and Switzerland. Native speakers from different regions and persons without medical backgrounds were equally important in drafting simple to understand text so that every patient should be able to fill out the questionnaires alone. Several specific examples demonstrated the importance of cross-regional language validation. Patients were also asked to give feedback and provide comments on every question, particularly if something was not clearly formulated or was missing. Every question of the final upper and lower German TESS versions was positively reviewed.

In conclusion, the German TESS versions for the upper and lower extremities are ready to use instruments for German-speaking countries, as well as with German-speaking individuals in other locations, to measure patient-reported physical functioning in patients treated with limb salvage surgery after benign and malignant bone and soft tissue tumors.

## Supplementary Information

Included is the original German version of the Toronto extremity salvage score.

This is the original German version of the Toronto extremity salvage score.
