# Supplementary material for: Cross-cultural adaption, translation and validation of the Toronto extremity salvage score (TESS) for patients in German-speaking countries
Source: Wien Klin Wochenschr. 2021 Apr 27;133(11-12):536–42. doi: 10.1007/s00508-021-01865-4 (PMC8195924; doi:10.1007/s00508-021-01865-4)
Supplement: Supplementary file 1 — Included is the original German version of the Toronto extremity salvage score. [file 508_2021_1865_MOESM1_ESM.pdf]

SS

# Radiation Therapy Oncology Group
## Phase II Soft Tissue Sarcoma
## Toronto Extremity Salvage Score (TESS)

RTOG Study 0630 Case #

PLACE LABEL HERE

Institution Institution No.

Participant's Initials Participant's I.D. No.

AMENDED DATA ☐ YES

**INSTRUCTIONS:** This sheet is the cover page used for submission of the (SAQ*-F) questionnaire. This page must be completed by the medical staff (nurse, data manager, physician, etc.) See detailed instructions for TESS questionnaire.

1 ☐ **TIME POINT**(1)

1 Baseline
2 12 months from start of treatment
3 18 months from start of treatment
4 24 months from start of treatment

2 ☐ **WAS PATIENT QUESTIONNAIRE COMPLETED?**(2)

1 No (Skip to question 3)
2 Yes

2A **DATE PATIENT QUESTIONNAIRE COMPLETED**

_____-_____-_____(3)

3 ☐ **REASON QUESTIONNAIRE WAS NOT COMPLETED**(4)

0 Not applicable, questionnaire was completed
1 Patient refused due to illness
2 Patient refused for other reason, specify__________________________(5)
3 Patient unable to be contacted
4 Institutional error
5 Tool not available in patient's language
6 Other reason, specify __________________________(6)
9 Unknown

4 ☐ **SPECIFY METHOD OF COMPLETION**(7)

0 Not applicable (not completed)
1 At appointment
2 By mail
3 By telephone
9 Unknown

5 ☐ **DID THE PATIENT REQUIRE ANY ASSISTANCE IN COMPLETING THE QUESTIONNAIRE?**(8)

0 Not applicable (not completed)
1 No
2 Yes
9 Unknown if assistance was given

6 ☐ **SPECIFY THE PERSON WHO ASSISTED THE PATIENT**(9)

0 Not applicable (not completed, no assistance)
1 Staff member
2 Family
3 Other, specify ____________________(10)
9 Unknown

7 ☐ **EXTENT OF ASSISTANCE GIVEN**(11)

0 Not applicable (not completed, no assistance)
1 Read items to patient
2 Interpreted items for patient
3 Marked items per patient's response
4 Combination of above, specify ________________________(12)
5 Other, specify ____________________(13)
9 Unknown

______________________________
**Signature of person completing this form**(14)

_____-_____-_____
**Date form completed**(15)

## Toronto Extremity Salvage Score (TESS)
## Administration of the Questionnaire

### General Guidelines

This questionnaire is designed as a measure of physical disability for patients undergoing limb salvage surgery for musculoskeletal tumours. It is a self-administered questionnaire.

There is an upper extremity and lower extremity version of the questionnaire. It is recommended that study personel complete the general information and review the instructions and sample questions with the subjects. The subjects can independently answer the remaining questions. Total completion time of the questionnaire averages 10 minutes.

### Scoring

Each question is a measure of the difficulty that the individual has performing the task. The total potential score for an item is a perfect performance score (ie. 5).

The scale has been designed to allow individuals to respond to a non-applicable category on an item if it is not something they perform in their everyday life. Consequently, a total questionnaire score, if desired, would be a standardized score ranging from 0 to 100 calculated by:

$$\frac{\text{sum of the item scores - \# items}}{\text{possible score range}} \times 100\%,$$

where, sum of the item scores = sum of difficulty responses
# items = items completed excluding the NA repsonse items
possible score range = (5 x #items) - (1 x #items)

### Mail Administration

The TESS questionnaires have been administered by mail and, although formal testing of measurement properties has not been undertaken, patients are able and willing to complete the forms and the scores fall within anticipated ranges.

SS

RTOG Study 0630 Case #

PLACE LABEL HERE

Institution Institution No.

Participant's Initials Participant's I.D. No.

## Toronto Extremity Salvage Score
## (Davis, 1996)

## Upper Extremity Questionnaire
## Lower Extremity Questionnaire

**Completed by patient at baseline, 12, 18, and 24 months from the start of treatment.**

**Note: only the upper or lower extremity form is completed, not both**

### TESS - UPPER EXTREMITY

Patient Study ID#: ________________ Patient Initials: ____________________
(first/middle/last)

Date of birth:______________________ Form Completion Date:____________________
(day/month/year) (day/month/year)

Months from start of treatment
- 0 Baseline
- 0 12 months
- 0 18 months
- 0 24 months

Site: 1____ Bone
2____ Soft Tissue

Side of Lesion: 1_____Right
2_____Left

Are you: 1_____right handed
2_____left handed

The following questions are about activities commonly performed in daily life. Each question asks that you mark each item (as in the examples below) opposite the description that best describes your ability to perform each task during the **past week.** Some activities will be extremely easy for you to do, others will be extremely difficult or impossible.

RTOG Study 0630 Case #

PLACE LABEL HERE

Institution Institution No.

Participant's Initials Participant's I.D. No.

**EXAMPLE**

Peeling vegetables is:

1____impossible to do.

2____extremely difficult.

3____moderately difficult.

4____a little bit difficult.

5____not at all difficult.

888____This task is not applicable for me.

You should choose the response "impossible to do...." if the activity is **something that you normally do** in your daily activities but are **now unable to do** because of physical limitations such as weakness, stiffness or pain.

If you do not perform an activity as part of your normal lifestyle you would choose the response "888" to indicate that the item is not applicable.

Mark all items ensuring that you choose the description that most accurately describes your abilities in the **past week.**

The following questions ask about your ability to perform activities that are common to everyday life. Considering the amount of difficulty you have performing the activity due to the current problem you are having with your arm, please answer the questions by choosing the answer that best describes your ability to do the activity **over the past week.**

**1) Putting on a pair of pants is:**

1____impossible to do.

2____extremely difficult.

3____moderately difficult.

4____a little bit difficult.

5____not at all difficult.

888____This task is not applicable for me.

**2) Tying shoe laces is:**

1____impossible to do.

2____extremely difficult.

3____moderately difficult.

4____a little bit difficult.

5____not at all difficult.

888____This task is not applicable for me.

**SS**

RTOG Study 0630 Case #

PLACE LABEL HERE

Institution Institution No.

Participant's Initials Participant's I.D. No.

**3) Putting on socks or stockings is:**

1____impossible to do.
2____extremely difficult.
3____moderately difficult.
4____a little bit difficult.
5____not at all difficult.

888____This task is not applicable for me.

**4) Showering is:**

1____impossible to do.
2____extremely difficult.
3____moderately difficult.
4____a little bit difficult.
5____not at all difficult.

888____This task is not applicable for me.

**5) Dressing my arms and upper body is:**

1____impossible to do.
2____extremely difficult.
3____moderately difficult.
4____a little bit difficult.
5____not at all difficult.

888____This task is not applicable for me.

**6) Buttoning a shirt is:**

1____impossible to do.
2____extremely difficult.
3____moderately difficult.
4____a little bit difficult.
5____not at all difficult.

888____This task is not applicable for me.

RTOG Study 0630 Case #

PLACE LABEL HERE

Institution Institution No.

Participant's Initials Participant's I.D. No.

**7) Tying a tie or a bow at the neck of a blouse is:**

1____impossible to do.
2____extremely difficult.
3____moderately difficult.
4____a little bit difficult.
5____not at all difficult.

888____This task is not applicable for me.

**8) Putting on make-up or shaving is:**

1____impossible to do.
2____extremely difficult.
3____moderately difficult.
4____a little bit difficult.
5____not at all difficult.

888____This task is not applicable for me.

**9) Brushing your teeth is:**

1____impossible to do.
2____extremely difficult.
3____moderately difficult.
4____a little bit difficult.
5____not at all difficult.

888____This task is not applicable for me.

**10) Brushing your hair is:**

1____impossible to do.
2____extremely difficult.
3____moderately difficult.
4____a little bit difficult.
5____not at all difficult.
888____This task is not applicable for me.

**11) Doing light household chores is:**

1____impossible to do.

2____extremely difficult.

3____moderately difficult.

4____a little bit difficult.

5____not at all difficult.

888____This task is not applicable for me.

**12) Gardening or yard work is:**

1____impossible to do.

2____extremely difficult.

3____moderately difficult.

4____a little bit difficult.

5____not at all difficult.

888____This task is not applicable for me.

**13) Preparing and serving meals is:**

1____impossible to do.

2____extremely difficult.

3____moderately difficult.

4____a little bit difficult.

5____not at all difficult.

888____This task is not applicable for me.

**14) Cutting food while eating is:**

1____impossible to do.

2____extremely difficult.

3____moderately difficult.

4____a little bit difficult.

5____not at all difficult.

888____This task is not applicable for me.

RTOG Study 0630 Case #

PLACE LABEL HERE

Institution Institution No.

Participant's Initials Participant's I.D. No.

**15) Drinking from a glass is:**

1____impossible to do.

2____extremely difficult.

3____moderately difficult.

4____a little bit difficult.

5____not at all difficult.

888____This task is not applicable for me.

**16) Performing heavy household chores is:**

1____impossible to do.

2____extremely difficult.

3____moderately difficult.

4____a little bit difficult.

5____not at all difficult.

888____This task is not applicable for me.

**17) Going shopping is:**

1____impossible to do.

2____extremely difficult.

3____moderately difficult.

4____a little bit difficult.

5____not at all difficult.

888____This task is not applicable for me.

**18) Giving or receiving change (ie. coins or bills) is:**

1____impossible to do.

2____extremely difficult.

3____moderately difficult.

4____a little bit difficult.

5____not at all difficult.

888____This task is not applicable for me.

RTOG Study 0630 Case #

PLACE LABEL HERE

Institution Institution No.

Participant's Initials Participant's I.D. No.

**19) Carrying a shopping bag or briefcase is:**

1____impossible to do.

2____extremely difficult.

3____moderately difficult.

4____a little bit difficult.

5____not at all difficult.

888____This task is not applicable for me.

**20) Lifting a box to an overhead shelf is:**

1____impossible to do.

2____extremely difficult.

3____moderately difficult.

4____a little bit difficult.

5____not at all difficult.

888____This task is not applicable for me.

**21) Turning a key in a lock is:**

1____impossible to do.

2____extremely difficult.

3____moderately difficult.

4____a little bit difficult.

5____not at all difficult.

888____This task is not applicable for me.

**22) Pushing or pulling open a door is:**

1____impossible to do.

2____extremely difficult.

3____moderately difficult.

4____a little bit difficult.

5____not at all difficult.

888____This task is not applicable for me.

**23) Writing is:**

1____impossible to do.

2____extremely difficult.

3____moderately difficult.

4____a little bit difficult.

5____not at all difficult.

888____This task is not applicable for me.

**24) Picking up small items is:**

1____impossible to do.

2____extremely difficult.

3____moderately difficult.

4____a little bit difficult.

5____not at all difficult.

888____This task is not applicable for me.

**25) Completing my usual duties at work is:** (Work includes a job outside the home or as a homemaker.)

1____impossible to do.

2____extremely difficult.

3____moderately difficult.

4____a little bit difficult.

5____not at all difficult.

888____This task is not applicable for me.

**26) Working my usual number of hours is:** (Working includes both a job outside the home and as a homemaker.)

1____impossible to do.

2____extremely difficult.

3____moderately difficult.

4____a little bit difficult.

5____not at all difficult.

888____This task is not applicable for me.

**27) Participating in my usual leisure activities is:**

1____impossible to do.

2____extremely difficult.

3____moderately difficult.

4____a little bit difficult.

5____not at all difficult.

888____This task is not applicable for me.

**28) Socializing with friends and family is:**

1____impossible to do.

2____extremely difficult.

3____moderately difficult.

4____a little bit difficult.

5____not at all difficult.

888____This task is not applicable for me.

**29) Participating in my usual sporting activities is:**

1____impossible to do.

2____extremely difficult.

3____moderately difficult.

4____a little bit difficult.

5____not at all difficult.

888____This task is not applicable for me.

**1) Considering all the activities in which I participate in daily life, I would rate my ability to perform these activities during the past week as:**

1______impossible to do.

2______extremely difficult.

3______moderately difficult.

4______a little bit difficult.

5______not at all difficult.

SS

RTOG Study 0630 Case #

PLACE LABEL HERE

Institution Institution No.

Participant's Initials Participant's I.D. No.

**2) I would rate myself as being :**

1______completely disabled

2______severely disabled.

3______moderately disabled.

4______mildly disabled.

5______not at all disabled.

Please comment below on any activities you find difficult to perform or on any other difficulties you experience due to the problem you currently have in your arm that you feel are important and have not been asked about in this questionnaire.

______________________________________________________________________

______________________________________________________________________

______________________________________________________________________

______________________________________________________________________

**Please check to make sure that you have not missed any questions.**

**Thank you for taking the time to answer these questions.**

RTOG Study 0630 Case #

PLACE LABEL HERE

Institution Institution No.

Participant's Initials Participant's I.D. No.

**TESS - LOWER EXTREMITY**

Patient Study ID#: ________________ Patient Initials: ____________________
(first/middle/last)

Date of birth:______________________ Form Completion Date:____________________
(day/month/year) (day/month/year)

Months from start of treatment
0 Baseline
0 12 months
0 18 months
0 24 months

Site: 1____ Bone
2____ Soft Tissue

The following questions are about activities commonly performed in daily life. Each question asks that you mark each item (as in the examples below) opposite the description that best describes your ability to perform each task during the **past week.** Some activities will be extremely easy for you to do, others will be extremely difficult or impossible.

**EXAMPLE**
Riding a bicycle is:

1____impossible to do.
2____extremely difficult.
3____moderately difficult.
4____a little bit difficult.
5____not at all difficult.

888____This task is not applicable for me.

You should choose the response "impossible to do...." if the activity is **something that you normally do** in your daily activities but are **now unable to do** because of physical limitations such as weakness, stiffness or pain.

If you do not perform an activity as part of your normal lifestyle you would choose the response "888" to indicate that the item is not applicable.
Mark all items ensuring that you choose the description that most accurately describes your abilities in the **past week.**

SS

RTOG Study 0630 Case #

PLACE LABEL HERE

Institution Institution No.

Participant's Initials Participant's I.D. No.

The following questions ask about your ability to perform activities that are common to every day life. Considering the amount of difficulty you have performing the activity due to the current problem you are having with your leg, please answer the questions by choosing the answer that best describes your ability to do the activity **over the past week.**

**1) Putting on a pair of pants is:**

1____impossible to do.
2____extremely difficult.
3____moderately difficult.
4____a little bit difficult.
5____not at all difficult.

888____This task is not applicable for me.

**2) Putting on shoes is:**

1____impossible to do.
2____extremely difficult.
3____moderately difficult.
4____a little bit difficult.
5____not at all difficult.

888____This task is not applicable for me.

**3) Putting on socks or stockings is:**

1____impossible to do.
2____extremely difficult.
3____moderately difficult.
4____a little bit difficult.
5____not at all difficult.

888____This task is not applicable for me.

**4) Showering is:**

1____impossible to do.
2____extremely difficult.
3____moderately difficult.
4____a little bit difficult.
5____not at all difficult.
888____This task is not applicable for me.

SS

RTOG Study 0630 Case #

PLACE LABEL HERE

Institution Institution No.

Participant's Initials Participant's I.D. No.

**5) Light household chores such as tidying and dusting are:**

1____impossible to do.

2____extremely difficult.

3____moderately difficult.

4____a little bit difficult.

5____not at all difficult.

888____This task is not applicable for me.

**6) Gardening and yard work are:**

1____impossible to do.

2____extremely difficult.

3____moderately difficult.

4____a little bit difficult.

5____not at all difficult.

888____This task is not applicable for me.

**7) Preparing and serving meals is:**

1____impossible to do.

2____extremely difficult.

3____moderately difficult.

4____a little bit difficult.

5____not at all difficult.

888____This task is not applicable for me.

**8) Going shopping is:**

1____impossible to do.

2____extremely difficult.

3____moderately difficult.

4____a little bit difficult.

5____not at all difficult.

888____This task is not applicable for me.

RTOG Study 0630 Case #

PLACE LABEL HERE

Institution Institution No.

Participant's Initials Participant's I.D. No.

**9) Heavy household chores such as vacuuming and moving furniture is:**

1____impossible to do.

2____extremely difficult.

3____moderately difficult.

4____a little bit difficult.

5____not at all difficult.

888____This task is not applicable for me.

**10) Getting in and out of the bath tub is:**

1____impossible to do.

2____extremely difficult.

3____moderately difficult.

4____a little bit difficult.

5____not at all difficult.

888____This task is not applicable for me.

**11) Getting out of bed is:**

1____impossible to do.

2____extremely difficult.

3____moderately difficult.

4____a little bit difficult.

5____not at all difficult.

888____This task is not applicable for me.

**12) Rising from a chair is:**

1____impossible to do.

2____extremely difficult.

3____moderately difficult.

4____a little bit difficult.

5____not at all difficult.

888____This task is not applicable for me.

**13) Kneeling is:**

1____impossible to do.

2____extremely difficult.

3____moderately difficult.

4____a little bit difficult.

5____not at all difficult.

888____This task is not applicable for me.

**14) Bending to pick something up off the floor is:**

1____impossible to do.

2____extremely difficult.

3____moderately difficult.

4____a little bit difficult.

5____not at all difficult.

888____This task is not applicable for me.

**15) Walking upstairs is:**

1____impossible to do.

2____extremely difficult.

3____moderately difficult.

4____a little bit difficult.

5____not at all difficult.

888____This task is not applicable for me.

**16) Walking downstairs is:**

1____impossible to do.

2____extremely difficult.

3____moderately difficult.

4____a little bit difficult.

5____not at all difficult.

888____This task is not applicable for me.

RTOG Study 0630 Case #

PLACE LABEL HERE

Institution Institution No.

Participant's Initials Participant's I.D. No.

**17) Driving is:**

1____impossible to do.

2____extremely difficult.

3____moderately difficult.

4____a little bit difficult.

5____not at all difficult.

888____This task is not applicable for me.

**18) Walking within the house is:**

1____impossible to do.

2____extremely difficult.

3____moderately difficult.

4____a little bit difficult.

5____not at all difficult.

888____This task is not applicable for me.

**9) Walking outdoors is:**

1____impossible to do.

2____extremely difficult.

3____moderately difficult.

4____a little bit difficult.

5____not at all difficult.

888____This task is not applicable for me.

**20) Sitting is:**

1____impossible to do.

2____extremely difficult.

3____moderately difficult.

4____a little bit difficult.

5____not at all difficult.

888____This task is not applicable for me.

RTOG Study 0630 Case #

PLACE LABEL HERE

Institution Institution No.

Participant's Initials Participant's I.D. No.

**21) Walking up or down hills or a ramp is:**

1____impossible to do.

2____extremely difficult.

3____moderately difficult.

4____a little bit difficult.

5____not at all difficult.

888____This task is not applicable for me.

**22) Standing upright is:**

1____impossible to do.

2____extremely difficult.

3____moderately difficult.

4____a little bit difficult.

5____not at all difficult.

888____This task is not applicable for me.

**23) Getting up from kneeling is:**

1____impossible to do.

2____extremely difficult.

3____moderately difficult.

4____a little bit difficult.

5____not at all difficult.

888____This task is not applicable for me.

**24) Getting in and out of a car is:**

1____impossible to do.

2____extremely difficult.

3____moderately difficult.

4____a little bit difficult.

5____not at all difficult.

888____This task is not applicable for me.

SS

RTOG Study 0630 Case #

PLACE LABEL HERE

Institution Institution No.

Participant's Initials Participant's I.D. No.

**25) Participating in sexual activities is:**

1____impossible to do.

2____extremely difficult.

3____moderately difficult.

4____a little bit difficult.

5____not at all difficult.

888____This task is not applicable for me.

**26) Completing my usual duties at work is:** (Work includes both a job outside the home and as a homemaker.)

1____impossible to do.

2____extremely difficult.

3____moderately difficult.

4____a little bit difficult.

5____not at all difficult.

888____This task is not applicable for me.

**27) Working my usual number of hours is:** (Working includes both a job outside the home and as a homemaker.)

1____impossible to do.

2____extremely difficult.

3____moderately difficult.

4____a little bit difficult.

5____not at all difficult.

888____This task is not applicable for me.

**28) Participating in my usual leisure activities is:**

1____impossible to do.

2____extremely difficult.

3____moderately difficult.

4____a little bit difficult.

5____not at all difficult.

888____This task is not applicable for me.

SS

RTOG Study 0630 Case #

PLACE LABEL HERE

Institution Institution No.

Participant's Initials Participant's I.D. No.

**29) Socializing with friends and family is:**

1____impossible to do.

2____extremely difficult.

3____moderately difficult.

4____a little bit difficult.

5____not at all difficult.

888____This task is not applicable for me.

**30) Participating in my usual sporting activities is:**

1____impossible to do.

2____extremely difficult.

3____moderately difficult.

4____a little bit difficult.

5____not at all difficult.

888____This task is not applicable for me.

**1) Considering all the activities in which I participate in daily life, I would rate the ability to perform these activities during the past week as:**

1_____impossible to do.

2_____extremely difficult.

3_____moderately difficult.

4_____a little bit difficult.

5_____not at all difficult.

**2) I would rate myself as being :**

1_____completely disabled

2_____severely disabled.

3_____moderately disabled.

4_____mildly disabled.

5_____not at all disabled.

Please comment below on any activities you find difficult to perform or on any other difficulties you experience due to the problem you currently have in your leg that you feel are important and have not been asked about in this questionnaire.

______________________________________________

______________________________________________

______________________________________________

**Please check to make sure that you have answered all the questions.**

**Thank you for taking the time to answer these questions.**
